# Carbon dioxide binary crystals *via* the thermal decomposition of RDX at high pressure†
†Electronic supplementary information (ESI) available: Experimental methods for the low temperature and high pressure growth and data collection. Theoretical methods are outlined together with the validation of the cluster expansion methodology. Crystallographic data tables are included. Raw data can be found at University of Strathclyde-Pure. See DOI: 10.1039/c7sc01379e



**DOI:** 10.1039/c7sc01379e

**Published:** 2017-05-04

**Authors:** L. E. Connor, C. A. Morrison, I. D. H. Oswald, C. R. Pulham, M. R. Warren

**Affiliations:** a Strathclyde Institute of Pharmacy and Biomedical Sciences , University of Strathclyde , 161 Cathedral Street , Glasgow , G4 0RE , UK . Email: iain.oswald@strath.ac.uk; b School of Chemistry , Centre for Science at Extreme Conditions , The University of Edinburgh , King's Buildings , David Brewster Road , Edinburgh EH9 3FJ , UK; c Diamond Light Source , Harwell Science and Innovation Campus , Didcot , Oxfordshire OX11 0DE , UK

## Abstract

A carbon dioxide and nitrous oxide solid solution has been captured in a diamond anvil cell following the thermal decomposition of 1,3,5-trinitroperhydro-1,3,5-triazine (RDX) at high temperatures and pressures. This is the first time a carbon dioxide binary solid has been observed at high pressure. This observation has stimulated low temperature crystallographic studies of this binary system using recently developed gas absorption apparatus and computational modelling.

## Abstract 2

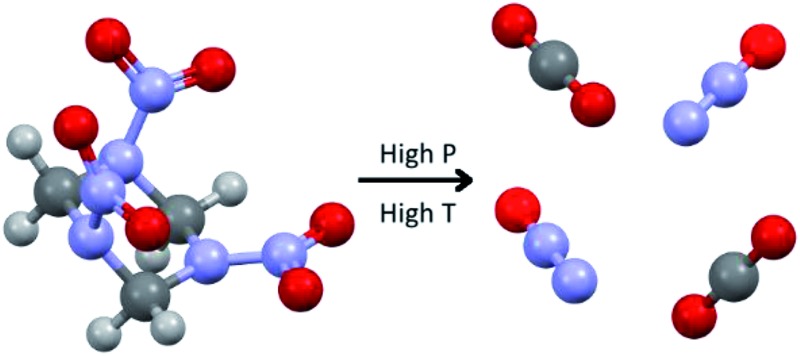
Binary crystals of carbon dioxide and nitrous oxide have been created from the decomposition of RDX.

## 

The study of energetic materials (explosives, propellants, gas generators) in the solid state under extremes of temperature and pressure are key to understanding the changes that may occur under operational conditions.^1^ HMX, FOX-7 and CL-20, as well as high-energy simple salts, have all been investigated under high pressure conditions.^2–7^

One material that has received a great deal of interest is 1,3,5-trinitroperhydro-1,3,5-triazine (RDX).^6,8–15^ It shows a rich phase behaviour under high pressures and temperatures; five polymorphs have been documented, with only the δ-polymorph remaining structurally uncharacterised. Hunter *et al.* provided a comprehensive overview of the solid-state chemistry of RDX using computational (dispersion-corrected density functional theory, DFT-D) methods to describe previously determined experimental results, as well as interpreting inelastic neutron scattering measurements as part of the study.^4^ Good agreement was found between the model and the experimental findings, giving confidence in the calculated vibrational properties and the changes in heat capacities of each phase as a function of temperature.

Investigating solid-state structures of high energy materials is important to better understand the processes of deflagration and detonation, but this is only part of the process. The thermal decomposition of RDX into reaction products is an area of intense research, with modelling playing an important role due to the very short timescales involved and the small quantities of product available for detection which hinder experimental characterisation. The decomposition of RDX has been investigated by a number of groups using both experimental and theoretical methods, with mass spectrometry and vibrational spectroscopy being utilised for product identification, as well as for the derivation of kinetic information.^13,16–21^ These studies, which were conducted under a variety of ambient- and high-pressure environments, discuss a number of decomposition mechanisms that RDX may undergo; these include the loss of NO_2_ groups,^21^ the cleavage of the C–N bond to create CH_2_–N_2_–O_2_ species, as well as whether the decomposition is a unimolecular or bimolecular process.^3^ Of particular interest to the current study is an article by Oyumi and Brill^13^ who showed that under slight increases in gas pressure (1–69 bar) there was a 4-fold increase in the production of CO_2_, suggesting that under higher pressure the formation of CO_2_ was favourable; the production of N_2_O remained constant irrespective of the gas pressure.

In this paper we first describe the capture of reaction products of RDX decomposition at high pressure and their subsequent characterisation using X-ray diffraction and spectroscopic methods. The second half of the paper then describes low temperature work on binary gas mixtures which were inspired by the high pressure work. In this section we have utilised developments in equipment for gas absorption studies to great effect in the crystallisation of binary gas mixtures, with additional insight into the crystallographic packing observed provided by computational modelling using DFT-D.

During the course of our investigations into the high-pressure, high-temperature polymorph of RDX (ε-form), the phase diagram indicated that conditions of 5.0 GPa and 548 K were sufficiently far from any phase boundaries to isolate the desired phase. These conditions are, however, very close to the region of the ε-form melting curve.^24^ After holding the sample at this temperature and pressure for 30 minutes, the powder showed signs of a transformation, with the diffraction pattern becoming more single-crystal like. To facilitate this change, the temperature was raised to 553 K and the sample maintained at this temperature for one hour. On cooling it was observed that the sample had become largely optically transparent with small yellow deposits at the side of the chamber, and the pressure inside the cell had substantially reduced to 3 GPa. X-ray powder diffraction patterns showed that this was a single crystal of a material that possessed a small unit cell, which was subsequently confirmed through data collection on the single crystal. The unit cell parameters observed were similar, but not identical to that expected for carbon dioxide (form I). Raman spectra of the single crystal showed peaks that could be attributed to carbon dioxide, but also peaks that could be attributed to nitrous oxide (Fig. 1). Nitrous oxide is isoelectronic with carbon dioxide and the crystal structures are also isostructural up to 5 GPa, with the lattice parameter of nitrous oxide being slightly greater than that of carbon dioxide (see Fig. 2) – above 5 GPa a transition to an orthorhombic phase has been observed for pure nitrous oxide.^25^ Hence our hypothesis was that a solid solution could be formed between these two compounds.

**Fig. 1:**
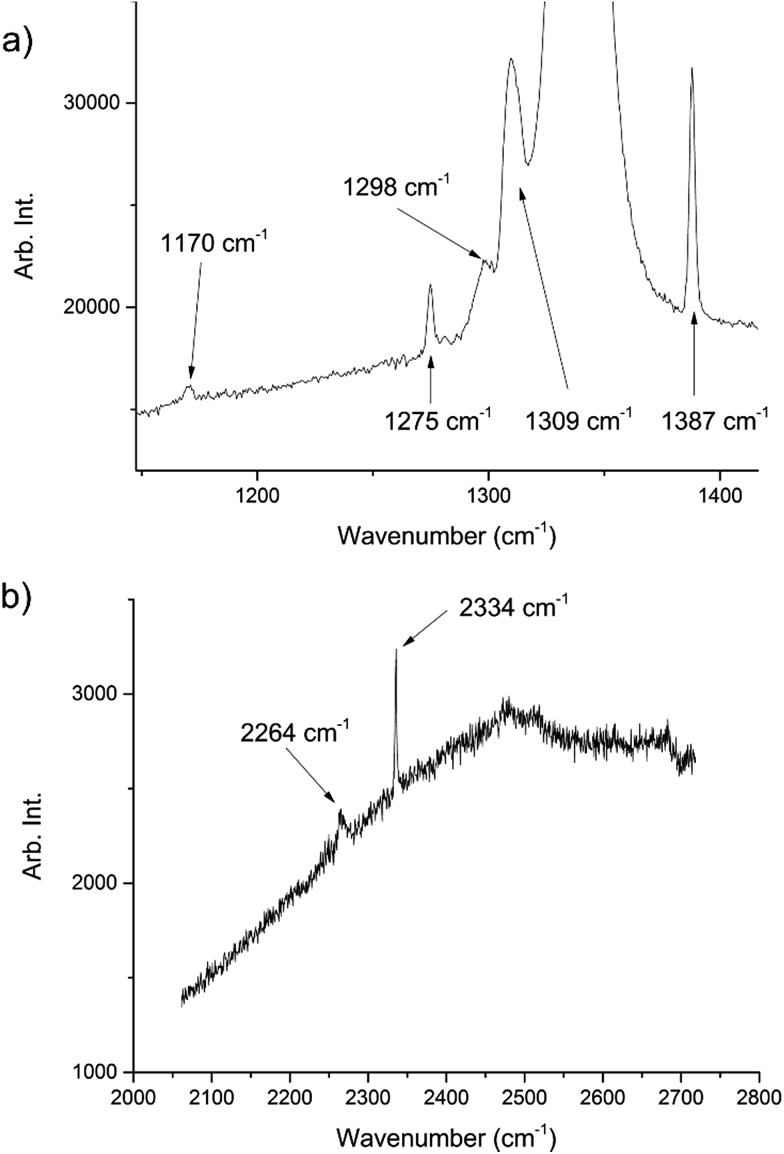
Raman spectra of the decomposition products showing peaks associated with both CO_2_ and N_2_O. The peaks at 1275, 1309, 1387, 2334 cm^–1^ are representative of CO_2_ (ref. 22) whilst those at 1170, 1298, 2264 cm^–1^ are representative of N_2_O.^23^ The large peak at ∼1350 cm^–1^ is from the diamond anvils. The high fluorescence can be attributed to the diamond anvils however there may have been contributions from residual yellow reaction deposits present in the cell.

**Fig. 2:**
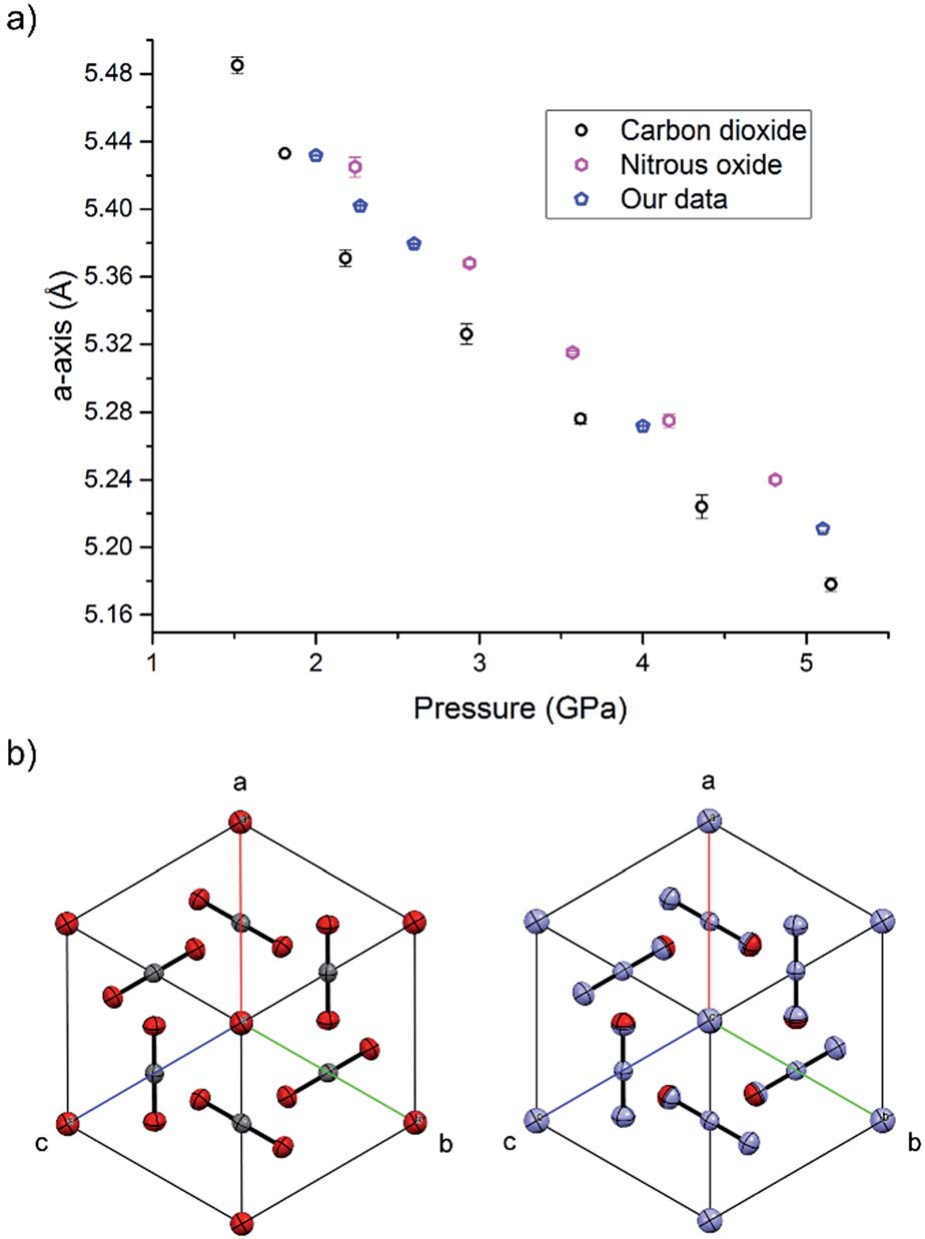
(a) The compression of the solid solution of carbon oxide and nitrous oxide at ambient temperature, together with values of previous studies of the pure components.^25,33^ (b) The isostructurality of carbon dioxide (left) and nitrous oxide (right) looking down the [111] direction.

High pressure X-ray diffraction studies were performed on the single crystal of the binary solid. Compression of the crystal showed that the lattice parameters lie between those of pure CO_2_ and N_2_O at pressures up to 5.1 GPa, and no changes in the crystal packing structure was observed (Fig. 2). Refinement of a disordered model at the different pressures suggested that the composition of the material was 70 mol% N_2_O with other reaction products contained in the uncharacterised yellow deposit. It is possible that other gaseous products were created in insufficient quantities to be determined however a smell of ammonia was observed on opening the cell. The products from this decomposition may be different to those obtained *via* other routes, *e.g.* shockwave, however the larger quantity of carbon dioxide is consistent with the observations of Oyumi and Brill^13^ albeit at much higher pressures.

A 3-term Vinet equation of state was used to determine the bulk modulus (*B*_0_) and pressure derivative (*B*′). The lack of data to low pressure caused a problem in determining the ambient pressure volume (*V*_0_) and the *B*_0_ and *B*′ values with sufficient precision hence we have determined two scenarios based on setting the *V*_0_ to specific values based on molecular volumes of CO_2_ and the hypothetical value from Mills *et al.* Table 1 shows these values together with determinations for CO_2_ (ref. 26) and our determination of N_2_O using the data of Mills *et al.* ^25^ It is worthy of note that recently Heit *et al.* have investigated the effects of the addition of thermal expansion to predicted compression behaviour in carbon dioxide and related these to experimental studies.^27^

**Table 1:** Parameters for the Vinet EOS^28^ obtained from Giordano *et al.* (CO_2_)^26^ and by fitting our data and the literature data for N_2_O.^25^ For the fitting of N_2_O data hypothetical values for *V*_0_, obtained from Mill *et al.*, are presented and left unrefined. Two fittings of the data for the binary crystal are presented using two values of *V*_0_, one of CO_2_ and the other of N_2_O

Compound	*V*_0_ (Å^3^ per molecule)	*B*_0_ (GPa)	*B*′
CO_2_ (ref. 26)	50(2)	3(1)	8.4(8)
CO_2_/N_2_O	50	3.66(18)	8.4(3)
CO_2_/N_2_O	48.6	5.1(2)	7.5(3)
N_2_O	48.6 (ref. 25)	6.0(3)	6.7(4)

Crystallisation of liquids and gases under extreme pressures (0–50 GPa) using diamond anvil cells (DAC) have been extensively used to map out phase diagrams which has led to numerous high profile publications.^29,30^ Included in these studies are reports of the binary phase diagrams of carbon dioxide with helium and noble gases. For both the He/CO_2_ and Ne/CO_2_ systems, the CO_2_ solidifies at ∼0.8 GPa leaving a He or Ne-rich fluid that subsequently solidifies as pure He or Ne at 11.6 GPa and 5.15 GPa, respectively, leading the authors to note that no compound or alloy of carbon dioxide has been observed.^31^

In the current work, the isostructural nature of CO_2_ and N_2_O has aided the formation of a stable binary crystal at high pressure. In the second half of this study we have explored the effects of low temperature conditions on this system but at ambient pressure. This augments a study by Solodovnik in which he observed these CO_2_–N_2_O systems as powders at very low temperatures (5–65 K) *via* transmission high energy electron diffraction measurements.^32^

To enable the low-temperature study, a recently developed gas cell (Fig. 3) was used to enable *in situ* control over the gas loading. The recent surge in studies of metal–organic frameworks (MOFs) and the need for structural characterisation of these materials under various gas-loadings has necessitated the design of a gas cell that could house crystals of MOFs and withstand positive pressures of gases whilst collecting diffraction data.^34^ The cell comprises a central union tee with 1/8, 1/8, 1/16 inch connections (Swagelok SS-200-3-2-1). The tee is connected to a standard goniometer head giving *x*, *y* and *z* translation, a 1/16 flexible PFA tubing (Cole-Parmer WZ-06407-41) to the gas control rig and a 0.3 mm glass quartz sample capillary with 10 μm wall thickness (Hampton research HR6-132, OD 0.3 mm, ID 0.28 mm, L 25 mm). Precise control over gas flow to the cell is provided by a series of pneumatic valves and alicat mass flow controllers (MFC's) (MC-200SCCM-D) allowing accurate quantities, pressure and mixtures of gases to be selected; this is ideal for the proposed experiments to investigate binary mixtures.

**Fig. 3:**
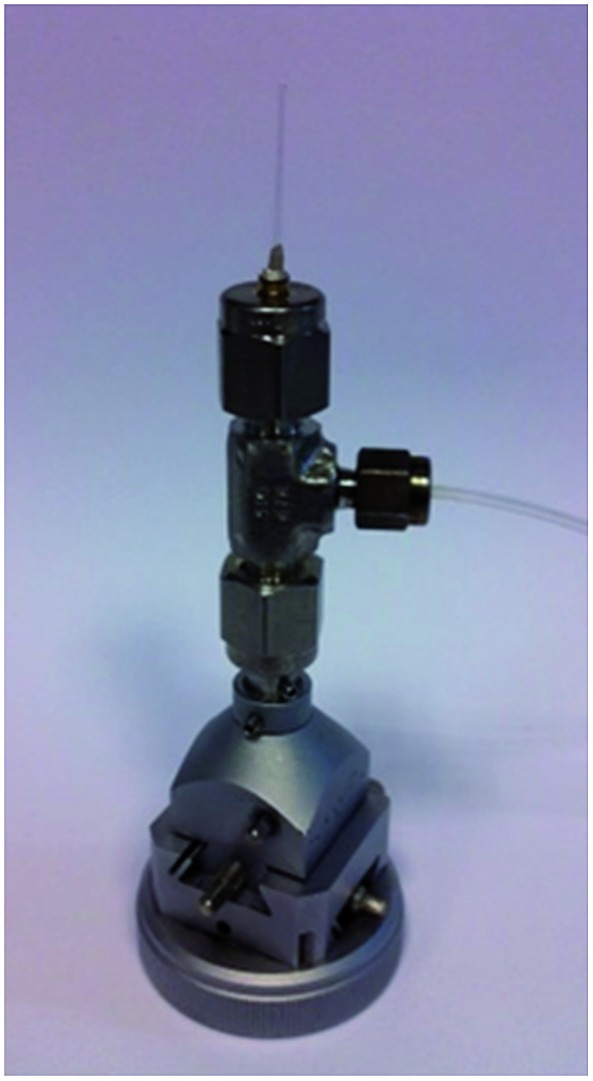
A photograph of the gas cell used in the low temperature experiment.

Previous work in the late 1990s and 2000s demonstrated the use of low-temperature devices to study the crystallisation of liquids under reduced temperatures in small capillaries.^35–46^ Crystallisation is induced by the reduction of temperature below the melting point or the shock freezing of the liquid by the application of liquid nitrogen to the outside of the capillary. Either temperature cycling or heating with an infrared laser warms the polycrystalline mass sufficiently to anneal into a single crystal or an oligo-crystal with domains large enough for structural solution and refinement.^46^ This technique has been extended further to investigate mixtures of liquids and gases with the notable research paper by Kirchner *et al.* on gas hydrates where the capillaries were immersed in liquid nitrogen to freeze the water and gas of choice before flame sealing and mounting onto the diffractometer.^47–50^ Using the new gas cell, the difficulties associated with the loading of capillaries are readily overcome, thereby offering a capability for exploration of a much wider range of systems.

For the crystallisation of the solid solution to have a successful outcome, care was needed to ensure that the gas pressure used to pressurise the gas cell was suitable to crystallise both compounds at the same temperature, as well as being within the pressure limits of the cell itself. If this pressure was not considered then there was a possibility that one component could crystallise before the other. From the comparison of the phase diagrams of the individual gases, a temperature of 185 K and a pressure of 1.5 bar was selected for crystallisation of both components to coincide. As an initial experiment the system was tested on the pure components alone. Before the gas was added to the cell, the cell was purged completely before being refilled with CO_2_ at a temperature higher than its melting point, which allowed the gas to equilibrate throughout the system. After a period of ten minutes the gas was cooled to *ca.* 200 K when liquid appeared in the cell. A number of trial runs ensued whereupon supercooling of the liquid was found to be a consistent problem due to the lack of nucleation points within the smooth capillary; this mirrors the problems observed for the crystallisation of liquids in previous work.^42^ At ∼184 K the liquid solidified into a polycrystalline mass. From this point the aim was to anneal the solid into one single crystal or, if this proved impossible, a manageable oligocrystal.

In this study we varied the temperature from 185 K to 200 K over a period of one hour (equating to 6 heat/cool cycles at 120 K per hour), which was sufficient to anneal a crystal for the diffraction studies (Fig. 4a). Variable temperature datasets of CO_2_ were then collected from 180 K to 100 K in 8 K steps, to provide a detailed unit cell volume/temperature graph for subsequent comparison against the binary-component samples. This data is presented in Fig. 4b, where a gradual and uniform reduction in the volume on cooling was observed, equating to a 5% reduction in cell volume over the temperature range explored.

**Fig. 4:**
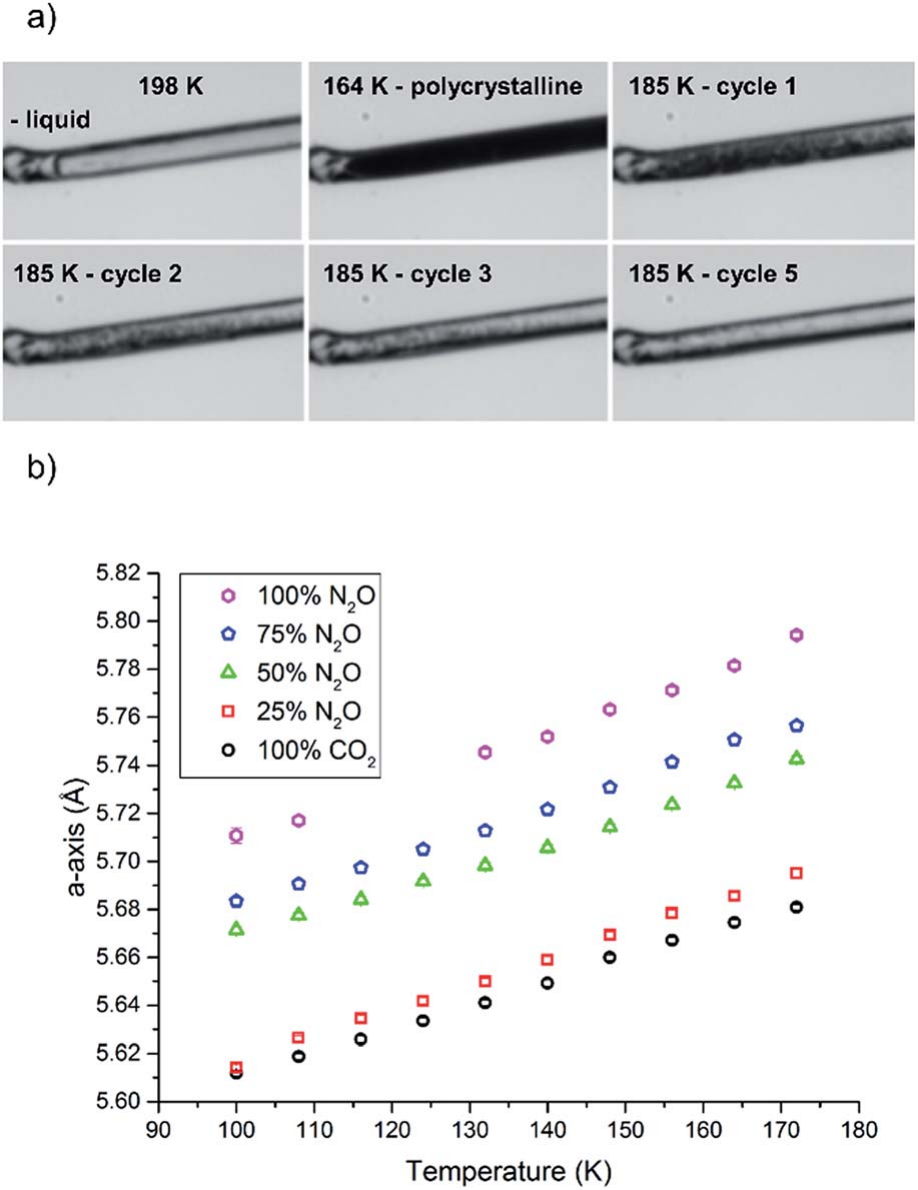
(a) Condensing and annealing crystallisation of 50 : 50 mixture of CO_2_/N_2_O; cycle 4 was omitted as it did not show a substantial visual change from cycle 3. (b) Unit cell parameters for various ratios of solid solutions as a function of temperature.

A similar procedure was then applied to investigate the crystallisation of pure N_2_O, using a gas pressure to give a similar melting point to CO_2_, such that that N_2_O began to liquefy at approximately the same temperature (201 K). However, N_2_O supercooled to a greater extent than CO_2_ and crystallised at 165 K. A similar temperature cycling regime was then performed to yield an oligocrystal (10 heat-cool cycles from 170 K to 185 K at 120 K min^–1^ with a pause for 2½ minutes before cooling). Variable temperature diffraction data were collected at the same temperatures as for the CO_2_ study. Unfortunately, during this data collection the synchrotron lost intensity, and so the datasets at 124 and 116 K were not recorded. Nevertheless the data at lower temperatures follow a consistent trend with the rest of the higher temperature data points obtained in this study.

The procedure was adjusted slightly for the binary mixtures. As with the previous samples the gas pressure that was chosen was 1.5 bar, but prior to sample loading the pressure system was completely evacuated (at 300 K) using a 10^–5^ mbar Turbo-pump for 20 minutes. Once the vacuum was achieved the cell was closed off so that the gases would be able to mix sufficiently in the feed pipes before being drawn into the narrow capillary. The system was filled with a 50 : 50 mixture of N_2_O and CO_2_ by ensuring the flow rate from each cylinder was set to be the same (5 mL min^–1^). After an equilibration period of 10 minutes the capillary was opened to the gas mixture. On cooling, the solution of N_2_O and CO_2_ began to condense at 198 K before supercooling to 164 K where it crystallised (Fig. 4a). Temperature annealing was performed between 170 K and 185 K with six cycles after which we had obtained a solid composed of a few crystals. From the unit cell parameters it is apparent that the crystal that was produced reflected a composition that was offset from the initial 50 mol% of each component. On average over the temperature series, refinement of the structure indicated that the crystal was composed of 34.9(6) mol% CO_2_ (green triangles Fig. 4b). To stabilise the refinement distance restraints were applied with thermal equivalency between each component. These were necessary as not only does the crystal have two components, the N_2_O is necessarily disordered over the inversion centre. This highlights one of the dangers of the annealing process in that it is a purification technique, hence despite starting with an even mixture, the resulting solid may not reflect the initial composition. Powder patterns were not collected for the starting material as it was very difficult to obtain a powder of sufficient quality to allow Pawley fitting.

For the two other compositions a similar methodology was employed, where the input of the gas into the pressure system was regulated to fixed values pertaining to 75 mol% N_2_O & 25 mol% CO_2_ and 25 mol% N_2_O & 75 mol% CO_2_. The system was, again, allowed to equilibrate before entering into the capillary. The condensation temperatures in both these experiments were similar to the previous experiments (200 K for 75 mol% N_2_O and 196 K for 25 mol% N_2_O). The annealing process (10 heat/cool cycles between 175 K and 188 K) for 75 mol% N_2_O crystals worked well with only a few crystallites left after the process that could be easily separated and treated as single crystals during data reduction procedures. The refinement of the structures in this series gave an average occupancy of 85.5(15) mol% (N_2_O). The 25 mol% N_2_O system was observed to be far more difficult to anneal which may be due to the temperatures selected for the annealing process, *i.e.* the upper temperature was not set high enough to allow the smaller crystallites to melt before cooling (188 K). Nevertheless we were able to identify the unit cell parameters from the diffraction pattern of the oligo-crystal. Using the previous model we were able to refine the occupancies and find that the crystal was on average 17.2(15) mol% N_2_O.

Overall the contraction of the unit cell parameters are linear with all fits having an *R* ^2^ value of 0.99. Thus we observe that the formation of the binary crystal does not seem to impact on the cooling characteristics of the solid, with an approximately 5% reduction in unit cell volume on cooling occurring in all samples. Each system does seem to show slight variation in the temperature at which it condenses and crystallises but the latter characteristic is notoriously difficult to control and predict due to the stochastic nature of nucleation; the smooth internal walls of the capillary provided few sites for nucleation.

To understand the lattice energies of the different systems and explain the disparity in the input and resulting stoichiometries we turned to computational modelling. The question that was posed was whether a thermodynamic driver existed for the adoption of the pure compounds over the mixed crystal, and whether the system was fully disordered or if it adopted homogenous regions. A full account of the computational methods employed can be found in the ESI.† Initially, the structure was expanded to a 2 × 2 × 2 primitive supercell, thus providing a periodic boundary condition model comprising 16 independent molecules, which was then studied using the CASTEP 8.0 code.^51^ The first task was to calculate the lattice energies of the pure compounds themselves which, for CO_2_, is straight-forward due to the molecular symmetry ‘fitting’ with the crystal symmetry *i.e.* carbon dioxide sits on the inversion fully ordered, hence all the molecules were ordered. For nitrous oxide, the construction of the lattice was complicated by the reduced molecular symmetry, however the reduction to the primitive symmetry cell setting allowed us to use a number of different orientations of N_2_O to calculate the lattice energy. The energies of models with various orientations of N_2_O were all within 3.5 kJ mol^–1^ of each other, so the molecular orientation does not seem to have a significant effect on the overall crystal packing energy. From these initial calculations the CO_2_ crystal structure is approximately 50 kJ mol^–1^ more thermodynamically stable than the N_2_O crystal structure (Fig. 5a). Identifying suitable models for the binary mixtures requires the disorder, both in terms of the identity of the molecule occupying the crystallographic site, and in the case of N_2_O the molecular orientation, to be taken into account. To allow the first disorder problem to be tackled in an efficient way, we turned to the use of a cluster expansion Hamiltonian using the 50 : 50 mixture as a starting point. This type of expansion allowed us to quickly search all possible packing arrangements and provide relative energies of each solution. To enable this, a training set of ten explicitly defined models that comprised equal occupancy of CO_2_ and N_2_O were defined and subjected to full DFT-D geometry optimisations. This data was then used to fit the parameters of the Hamilton presented in eqn (1). Here the total energy (*E*_total_) of the unit cell is expressed in terms of the identity of nearest neighbouring contacts, for 16 independent molecules that have 12 nearest neighbours (in a face-centred cubic arrangement).1
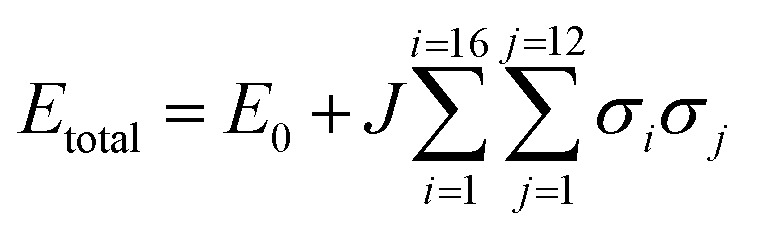



**Fig. 5:**
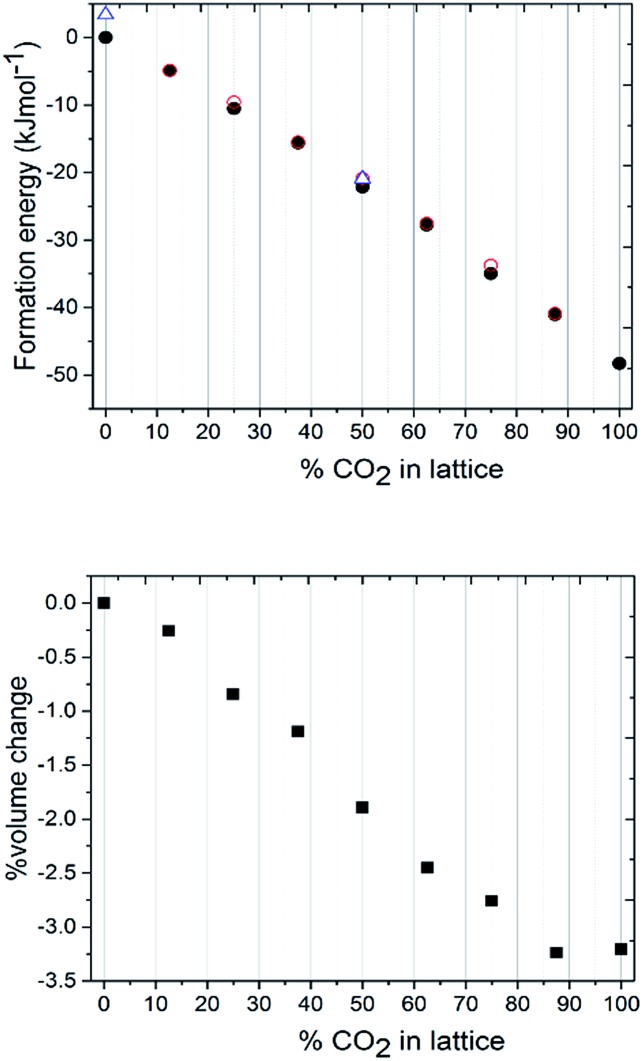
(a) Graph of relative lattice energy *versus* % CO_2_ doping in N_2_O crystal lattice, as obtained by DFT-D. Open red circles: most heterogeneously packed lattice, filled black circles: most homogeneously packed lattice. Open blue triangles: half of the N_2_O molecules from the lowest energy lattice randomly switched with respect to terminal N/O identity. Bottom: Graph of percentage unit cell volume change with increasing CO_2_ doping in N_2_O crystal lattice.

Assigning *σ* in eqn (1) to the value of +1 sets the molecular identity for that molecule to CO_2_; *σ* set to –1 assigns the molecule to N_2_O. The Hamiltonian comprises just two parameters: *E*_0_ which is a baseline energy fitting term, and *J* which is a weighting coefficient. If *J* adopts a positive value heterogeneous neighbouring contacts are energetically preferred; a negative value indicates that homogeneous neighbours, or clustering of like-molecules, will lower the total energy of the lattice. Finally, the magnitude of *J* indicates the spread of total energies obtainable by varying *σ*_*i*_ *σ*_*j*_; a small value indicates little change in energy as the neighbour identities are swapped, which is indicative of a fully crystallographically disordered system. Values of *E*_0_ = –16226.5935(18) eV and *J* = –0.00018(2) were returned based on a least squares fit to the total energies and neighbouring contact information for the training set of models, as outlined in Table S7.† The comparison between the total energies predicted by the cluster expansion expression compared to the DFT-D energies is shown in Fig. S1.† From the least squares fit parameters it is apparent that the total energy of the crystallographic lattice can be minimised if nearest neighbour contacts are homogeneous, however, the small magnitude of *J* suggest that the range in total energy obtained by varying the identity of the neighbouring contacts is very small; this is readily observable form the raw DFT energies presented in Table S7,† where the energy separation observed for the two models in the training set that represent the extremes of highly heterogeneous and homogeneous packing is only 1.2 kJ mol^–1^. Thus it seems highly likely that the CO_2_/N_2_O co-crystallised system is a highly disordered system.

The close correlation between the cluster expansion Hamiltonian energies and the DFT energies gave confidence to use the Hamiltonian expression to systematically search through all possible variants of CO_2_/N_2_O packing in the larger 2 × 2 × 2 conventional unit cell setting, which comprised 32 independent molecules. Searching through all possible combinations of alternating +1 and –1 combinations generated no further structures which were lower in energy than those already located in the DFT-D training set.

The next stage of the computational modelling work involved systematically varying the ratio of CO_2_ to N_2_O in the unit cell. All calculations were performed at the DFT-D level on the 2 × 2 × 2 primitive unit cell. Calculations started with all 16 molecules assigned to N_2_O, and then proceeded in steady increments where the identity of two molecules were switched to CO_2_ until all had changed their assignment to CO_2_. The cluster expansion process was utilised for each model to construct two unit cells, comprising maximum and minimum clustering of like-molecules, in order that an upper and lower energy range for that binary crystal composition could be explored. The total energies obtained were then recast as crystal lattice energies, by subtracting the energies of the appropriate number of isolated CO_2_ and N_2_O molecular units, with the value obtained for the parent all-N_2_O structure set as the baseline. Note the further level of disorder which arises for N_2_O due to terminal N/O identity was also pursued by randomly swapping the orientation of half of the N_2_O molecules over with respect to the lowest energy structures obtained for the all-N_2_O lattice and the 50 : 50 CO_2_ : N_2_O lattice. Results are presented in Fig. 5a.

The most obvious feature of the data presented in Fig. 5a is that the crystal lattice energy steadily increases (*i.e.* gets more negative) as the level of CO_2_ doping increases. The energy change upon doping the crystal lattice with increasing amounts of CO_2_ far outweighs any variation in energy concerned with variation in CO_2_/N_2_O neighbour contact interactions, which never exceeds 1.6 kJ mol^–1^ for this 2 × 2 × 2 primitive lattice supercell, or for the effects of N_2_O molecular orientation disorder, which never exceeds 3.5 kJ mol^–1^. The percentage volume change upon CO_2_ doping shows an overall contraction of the unit cell, of up to 3% compared to values in the range of 5–6% for the experimentally determined structures. Note the ‘S’ type feature to this plot: the most significant changes in unit cell volume are observed when the heterogeneity of the crystal exceeds 20 mol%. It is therefore understandable from an energetics point of view that the ratios of each component captured in the single crystal being analysed may vary on annealing from the input ratio *i.e.* 25 mol% N_2_O resolving to 17 mol%. It should be noted that the data we collected were on one particular crystal in the capillary and that the composition of a different crystal or vapour would be more CO_2_-rich than would normally be expected; on choosing a viable crystal for diffraction we may have overlooked such crystals.

In conclusion we have been able to capture the reaction products from the thermal decomposition of RDX. These form a solid solution under high-pressure conditions, with the structural stability imparted due to the isostructural and isoelectronic nature of the two products CO_2_ and N_2_O. The solid solution shows a similar compressibility to both its pure components, but failure of the gasket limited our exploration of pressure beyond 5.1 GPa where nitrous oxide is known to undergo a phase transition. The choice of material for the gasket was not explored but may influence the products of the decomposition. The stable solid state behaviour continues at low temperature where we have observed a range of solid solutions enabled by the advancements in equipment for absorption studies. Theoretical calculations have shown that the binary solid is disordered with respect to molecular identity at the crystallographic sites and the molecular orientation of N_2_O, and that the crystal lattice energies are strengthened as more CO_2_ is doped into the lattice. This may account for the disparity between the initial gas composition and that identified by single crystal diffraction. This study opens up the possibilities for studying *in situ* gas mixtures and gas hydrates using fine control over input streams. Such studies are likely to be invaluable for the study of the chemistry in planetary science as well as extending the crystal engineering approaches to very simple molecules.
