# Supplementary material for: Carbon dioxide binary crystals via the thermal decomposition of RDX at high pressure
Source: Chem Sci. 2017 May 4;8(7):4872–8. doi: 10.1039/c7sc01379e (PMC5603901; doi:10.1039/c7sc01379e)
Supplement: Supplementary file 1 [file SC-008-C7SC01379E-s001.pdf]

Electronic Supplementary Material (ESI) for Chemical Science.
This journal is © The Royal Society of Chemistry 2017

Carbon dioxide binary crystals via the thermal decomposition of RDX at high pressure.

L.E. Connor,[a] C.A. Morrison,[b] I.D.H. Oswald,[a*] C.R. Pulham,[b] and M. Warren[c]

[a]Strathclyde Institute of Pharmacy and Biomedical Sciences, University of Strathclyde, 161 Cathedral Street, Glasgow, G4 0RE Email: iain.oswald@strath.ac.uk

[b]School of Chemistry and Centre for Science at Extreme Conditions, The University of Edinburgh, King's Buildings, David Brewster Road, Edinburgh EH9 3FJ, U.K.

[c]Diamond Light Source, Harwell Science and Innovation Campus, Didcot, Oxfordshire, OX11 0DE, U.K.

Crystallography

Low temperature

X-ray intensities were collected at station I19 at Diamond Light Source, UK. The wavelength used was 0.6889 Å. The data were collected on a Pilatus detector and converted using program cbf_to_sfrm developed by Johnson and Probert.[1] Data were processed using APEX 3 software with usual absorption correction (SADABS).[2] The structure was solved using ShelXT[3] and refined using full matrix least-squares on all data using OLEX2 software.[4] For carbon dioxide the refinement was straight forward with both atoms refined anisotropically. For nitrous oxide the refinement required the use of disorder components. The central nitrogen atom is fully ordered but the second nitrogen atom is 50% disordered with oxygen. The atom positions were restrained to be 1.13 Å for N-N distance and 1.19 Å for the N-O distance. The thermal parameters were constrained to be the same for both atoms. An extinction parameter was required in the refinement of these sets of structures. For the disordered models two parts were made from the CO_2 model and N_2O model. A free variable was set for the occupancy of these atoms and allowed to refine using the constraints and restraints outlined above with the addition of distance restraints of 1.16 Å for the C-O distance.

High pressure

High-pressure studies were performed using Merrill-Bassett diamond anvil cell (DAC) with 600 µm culet diamonds glued on tungsten carbide backing disks.[5] A 250 µm thick tungsten foil gasket was indented to ~90 µm, and a 300 µm hole drilled in the indented portion of the gasket to create a sample chamber. A powder of RDX was loaded into the cell together with a ruby to allow in-situ pressure measurement via the Ruby fluorescence technique.[6]

The pressure inside the cell was increased to 5 GPa and heated in an oven to 548 K for 30 minutes. After this period the X-ray diffraction pattern exhibited localised spots rather than powder rings (Daresbury station 9.1). The cell was heated further at 553 K for 60 minutes. The cell was cooled to room temperature and the pressure had dropped to 3.1 GPa and the contents were liquid with yellow product towards the side of the gasket. By varying the temperature with a hot-air gun a single crystal was grown and data collected.

X-ray intensities were collected on a Bruker APEX-2 diffractometer (λ = 0.71073 Å). The data were reduced using APEX 2 software using dynamic masking procedure as outlined in Dawson et al. Absorption corrections were performed using SADABS. Initial refinement parameters were taken from the low temperature 50:50 structure and used to refine against the data. This showed that a 70% N_2O solid solution with CO_2 was present.

Table S1. Experimental details

For all structures: M_r = 44.02, Cubic, $Pa\bar{3}$, Z = 2. Experiments were carried out at 298 K with Mo $K\alpha$ radiation using a Bruker SMART APEX II CCD area detector. Refinement was on 9 parameters with 3 restraints.

	comp01	comp02	comp03	comp04
Crystal data				
Chemical formula	0.69(N2O):0.31(CO_2)	0.66(N2O):0.34(CO_2)	0.70(N2O):0.3(CO_2)	0.72(N2O):0.28(CO_2)
a (Å)	5.4317 (4)	5.4018 (5)	5.3794 (5)	5.2718 (5)
V (Å^3)	160.25 (4)	157.62 (4)	155.67 (4)	146.51 (4)
μ (mm^{-1})	0.09	0.09	0.09	0.09
Crystal size (mm)	0.3 × 0.3 × 0.1	0.3 × 0.3 × 0.1	0.3 × 0.3 × 0.1	0.3 × 0.3 × 0.1
Data collection				
Absorption correction	Multi-scan *SADABS2004*/1 (Bruker,2004) was used for absorption correction. R(int) was 0.0476 before and 0.0214 after correction. The Ratio of minimum to maximum transmission is 0.6372. The λ/2 correction factor is 0.0015.	Multi-scan *SADABS2004*/1 (Bruker,2004) was used for absorption correction. R(int) was 0.0495 before and 0.0252 after correction. The Ratio of minimum to maximum transmission is 0.5883. The λ/2 correction factor is 0.0015.	Multi-scan *SADABS2004*/1 (Bruker,2004) was used for absorption correction. R(int) was 0.0418 before and 0.0217 after correction. The Ratio of minimum to maximum transmission is 0.6033. The λ/2 correction factor is 0.0015.	Multi-scan *SADABS2004*/1 (Bruker,2004) was used for absorption correction. R(int) was 0.0438 before and 0.0169 after correction. The Ratio of minimum to maximum transmission is 0.6581. The λ/2 correction factor is 0.0015.
T_{min}, T_{max}	0.637, 1	0.588, 1	0.603, 1	0.658, 1
No. of measured, independent and observed [$I > 2\sigma(I)$] reflections	780, 50, 42	770, 50, 40	756, 50, 40	373, 46, 34
R_{int}	0.021	0.023	0.021	0.018
(sin θ/λ)$_{max}$ (Å^{-1})	0.617	0.621	0.624	0.622
Refinement				
$R[F^2 > 2\sigma(F^2)]$, $wR(F^2)$, S	0.017, 0.043, 1.25	0.014, 0.032, 1.24	0.016, 0.032, 1.30	0.019, 0.044, 1.27
No. of reflections	50	50	50	46
$\Delta\rangle_{max}$, $\Delta\rangle_{min}$ (e Å^{-3})	0.02, -0.02	0.02, -0.02	0.02, -0.02	0.03, -0.03

	comp06
Crystal data	
Chemical formula	0.75(N2O):0.25(CO_2)
a (Å)	5.2109 (13)
V (Å^3)	141.49 (11)
μ (mm^{-1})	0.10
Crystal size (mm)	0.3 × 0.3 × 0.1
Data collection	
Absorption correction	Multi-scan<br>*SADABS2014*/5 (Bruker,2014/5) was used for absorption correction. wR2(int) was 0.0893 before and 0.0311 after correction. The Ratio of minimum to maximum transmission is 0.8092. The λ/2 correction factor is Not present.
T_{min}, T_{max}	0.603, 0.745
No. of measured, independent and observed [$I > 2\sigma(I)$] reflections	582, 47, 33
R_{int}	0.022
$(\sin \theta/\lambda)_{max}$ (Å^{-1})	0.622
Refinement	
$R[F^2 > 2\sigma(F^2)]$, $wR(F^2)$, S	0.023, 0.080, 1.34
No. of reflections	47
$\Delta\rangle_{max}$, $\Delta\rangle_{min}$ (e Å^{-3})	0.06, -0.07

Computer programs: *SAINT* v7.23A (Bruker, 2003), *SAINT* v7.34A (Bruker, 200?), XL (Sheldrick, 2008), Olex2 (Dolomanov *et al.*, 2009).

Table S2. Experimental details

For all structures: M_r = 44.01, Cubic, $Pa\bar{3}$, Z = 4. Experiments were carried out with Synchrotron radiation, λ = 0.6889 Å using a Kappa Rigaku Saturn724+. Refinement was on 6 parameters.

	02_172k_co2	03_164k_co2	04_156k_co2	05_148k_co2
Crystal data				
Chemical formula	CO_2	CO_2	CO_2	CO_2
a (Å)	5.6809 (11)	5.6745 (12)	5.6671 (12)	5.6599 (12)
V (Å^3)	183.34 (11)	182.72 (12)	182.00 (12)	181.31 (12)
μ (mm^{-1})	0.16	0.16	0.16	0.16
Crystal size (mm)	0.5 × 0.28 × 0.28	0.5 × 0.28 × 0.28	0.5 × 0.28 × 0.28	0.5 × 0.28 × 0.28
Data collection				
Absorption correction	Multi-scan *SADABS2014*/5 (Bruker,2014/5) was used for absorption correction. wR2(int) was 0.1125 before and 0.0484 after correction. The Ratio of minimum to maximum transmission is 0.7598. The λ/2 correction factor is 0.00150.	Multi-scan *SADABS2014*/5 (Bruker,2014/5) was used for absorption correction. wR2(int) was 0.1172 before and 0.0509 after correction. The Ratio of minimum to maximum transmission is 0.7679. The λ/2 correction factor is 0.00150.	Multi-scan *SADABS2014*/5 (Bruker,2014/5) was used for absorption correction. wR2(int) was 0.1424 before and 0.0792 after correction. The Ratio of minimum to maximum transmission is 0.5948. The λ/2 correction factor is 0.00150.	Multi-scan *SADABS2014*/5 (Bruker,2014/5) was used for absorption correction. wR2(int) was 0.1147 before and 0.0508 after correction. The Ratio of minimum to maximum transmission is 0.7598. The λ/2 correction factor is 0.00150.
T_{min}, T_{max}	0.567, 0.746	0.573, 0.746	0.444, 0.746	0.567, 0.746
No. of measured, independent and observed [$I > 2\sigma(I)$] reflections	2293, 110, 104	2293, 109, 103	2208, 107, 101	2241, 107, 103
R_{int}	0.029	0.029	0.050	0.032
$(\sin \theta/\lambda)_{max}$ (Å^{-1})	0.752	0.753	0.738	0.739
Refinement				
$R[F^2 > 2\sigma(F^2)]$, $wR(F^2)$, S	0.026, 0.072, 1.16	0.026, 0.066, 1.24	0.024, 0.065, 1.14	0.021, 0.053, 1.20
No. of reflections	110	109	107	107
$\Delta\rangle_{max}$, $\Delta\rangle_{min}$ (e Å^{-3})	0.11, -0.13	0.10, -0.13	0.07, -0.12	0.11, -0.11

	06_140k_co2	07_132k_co2	08_124k_co2	09_116k_co2
Crystal data				
Chemical formula	CO_2	CO_2	CO_2	CO_2
a (Å)	5.6492 (12)	5.6411 (12)	5.6335 (12)	5.6258 (12)
V (Å^3)	180.29 (11)	179.51 (11)	178.79 (11)	178.05 (11)
μ (mm^{-1})	0.16	0.16	0.16	0.16
Crystal size (mm)	0.5 × 0.28 × 0.28	0.5 × 0.28 × 0.28	0.5 × 0.28 × 0.28	0.5 × 0.28 × 0.28
Data collection				
Absorption correction	Multi-scan *SADABS2014*/5 (Bruker,2014/5) was used for absorption correction. wR2(int) was 0.1196 before and 0.0534 after correction. The Ratio of minimum to maximum transmission is 0.7456. The λ/2 correction factor is 0.00150.	Multi-scan *SADABS2014*/5 (Bruker,2014/5) was used for absorption correction. wR2(int) was 0.1446 before and 0.0821 after correction. The Ratio of minimum to maximum transmission is 0.6147. The λ/2 correction factor is 0.00150.	Multi-scan *SADABS2014*/5 (Bruker,2014/5) was used for absorption correction. wR2(int) was 0.1477 before and 0.0816 after correction. The Ratio of minimum to maximum transmission is 0.6065. The λ/2 correction factor is 0.00150.	Multi-scan *SADABS2014*/5 (Bruker,2014/5) was used for absorption correction. wR2(int) was 0.1428 before and 0.0802 after correction. The Ratio of minimum to maximum transmission is 0.5971. The λ/2 correction factor is 0.00150.
T_{min}, T_{max}	0.556, 0.746	0.459, 0.746	0.453, 0.746	0.446, 0.746
No. of measured, independent and observed [$I > 2\sigma(I)$] reflections	2238, 107, 103	2132, 107, 103	2120, 107, 101	2113, 107, 100
R_{int}	0.033	0.052	0.053	0.050
$(\sin \theta/\lambda)_{max}$ (Å^{-1})	0.741	0.742	0.743	0.744
Refinement				
$R[F^2 > 2\sigma(F^2)]$, $wR(F^2)$, S	0.022, 0.061, 1.23	0.022, 0.050, 1.25	0.023, 0.060, 1.24	0.023, 0.062, 1.16
No. of reflections	107	107	107	107
$\Delta\rangle_{max}$, $\Delta\rangle_{min}$ (e Å^{-3})	0.11, -0.13	0.10, -0.10	0.08, -0.13	0.10, -0.12

	10_108k_co2	11_100k_co2
Crystal data		
Chemical formula	CO_2	CO_2
a (Å)	5.6188 (12)	5.6119 (12)
V (Å^3)	177.39 (11)	176.74 (11)
μ (mm^{-1})	0.16	0.16
Crystal size (mm)	0.5 × 0.28 × 0.28	0.5 × 0.28 × 0.28
Data collection		
Absorption correction	Multi-scan *SADABS2014*/5 (Bruker,2014/5) was used for absorption correction. wR2(int) was 0.1420 before and 0.0779 after correction. The Ratio of minimum to maximum transmission is 0.6216. The λ/2 correction factor is 0.00150.	Multi-scan *SADABS2014*/5 (Bruker,2014/5) was used for absorption correction. wR2(int) was 0.1445 before and 0.0809 after correction. The Ratio of minimum to maximum transmission is 0.6689. The λ/2 correction factor is 0.00150.
T_{min}, T_{max}	0.464, 0.746	0.499, 0.746
No. of measured, independent and observed [$I > 2\sigma(I)$] reflections	2080, 107, 101	2060, 107, 100
R_{int}	0.051	0.053
$(\sin \theta/\lambda)_{max}$ (Å^{-1})	0.745	0.745
Refinement		
$R[F^2 > 2\sigma(F^2)]$, $wR(F^2)$, S	0.023, 0.059, 1.22	0.023, 0.057, 1.18
No. of reflections	107	107
$\Delta\rangle_{max}$, $\Delta\rangle_{min}$ (e Å^{-3})	0.09, -0.13	0.12, -0.13

Computer programs: *SAINT* v8.34A (Bruker, 2013), XT (Sheldrick, 2015), XL (Sheldrick, 2008), Olex2 (Dolomanov *et al.*, 2009).

Table S3. Experimental details

For all structures: N_2O, M_r = 44.02, Cubic, $Pa\bar{3}$, Z = 4. Experiments were carried out as detailed in Table S2. Refinement was with 2 restraints.

	02_172k_n20	03_164k_n2o	04_156k_n2o	05_148k_n2o
Crystal data				
Temperature (K)	172	164	156	148
a (Å)	5.7942 (13)	5.7815 (13)	5.7723 (13)	5.7596 (13)
V (Å^3)	194.53 (13)	193.25 (13)	192.33 (13)	191.06 (13)
μ (mm^{-1})	0.14	0.14	0.14	0.14
Crystal size (mm)	0.5 × 0.28 × 0.28	0.5 × 0.28 × 0.28	0.5 × 0.28 × 0.28	0.5 × 0.28 × 0.28
Data collection				
Absorption correction	Multi-scan *SADABS2014*/5 (Bruker,2014/5) was used for absorption correction. wR2(int) was 0.2015 before and 0.0881 after correction. The Ratio of minimum to maximum transmission is 0.4426. The λ/2 correction factor is 0.00150.	Multi-scan *SADABS2014*/5 (Bruker,2014/5) was used for absorption correction. wR2(int) was 0.1992 before and 0.0839 after correction. The Ratio of minimum to maximum transmission is 0.4221. The λ/2 correction factor is 0.00150.	Multi-scan *SADABS2014*/5 (Bruker,2014/5) was used for absorption correction. wR2(int) was 0.1951 before and 0.0837 after correction. The Ratio of minimum to maximum transmission is 0.4496. The λ/2 correction factor is 0.00150.	Multi-scan *SADABS2014*/5 (Bruker,2014/5) was used for absorption correction. wR2(int) was 0.1982 before and 0.0846 after correction. The Ratio of minimum to maximum transmission is 0.4634. The λ/2 correction factor is 0.00150.
T_{min}, T_{max}	0.330, 0.746	0.315, 0.746	0.336, 0.746	0.345, 0.745
No. of measured, independent and observed [$I > 2\sigma(I)$] reflections	2611, 115, 94	2257, 116, 110	2195, 116, 111	2196, 114, 107
R_{int}	0.057	0.057	0.057	0.056
(sin θ/λ)$_{max}$ (Å^{-1})	0.757	0.749	0.750	0.752
Refinement				
$R[F^2 > 2\sigma(F^2)]$, $wR(F^2)$, S	0.033, 0.099, 1.27	0.036, 0.087, 1.13	0.036, 0.075, 1.22	0.030, 0.078, 1.14
No. of reflections	115	116	116	114
No. of parameters	8	8	7	7
$\Delta\rangle_{max}$, $\Delta\rangle_{min}$ (e Å^{-3})	0.10, -0.11	0.11, -0.11	0.18, -0.17	0.12, -0.09

	06_140k_n2o	07_132k_n2o	10_108k_n2o	11_100k_n2o
Crystal data				
Temperature (K)	140	132	108	172
a (Å)	5.7505 (14)	5.7370 (14)	5.7191 (16)	5.711 (3)
V (Å^3)	190.16 (14)	188.82 (14)	187.06 (15)	186.2 (3)
μ (mm^{-1})	0.14	0.14	0.14	0.14
Crystal size (mm)	0.5 × 0.28 × 0.28	0.5 × 0.28 × 0.28	0.5 × 0.28 × 0.28	0.5 × 0.28 × 0.28
Data collection				
Absorption correction	Multi-scan *SADABS2014*/5 (Bruker,2014/5) was used for absorption correction. wR2(int) was 0.1978 before and 0.0884 after correction. The Ratio of minimum to maximum transmission is 0.3990. The λ/2 correction factor is 0.00150.	Multi-scan *SADABS2014*/5 (Bruker,2014/5) was used for absorption correction. wR2(int) was 0.1946 before and 0.0939 after correction. The Ratio of minimum to maximum transmission is 0.3671. The λ/2 correction factor is 0.00150.	Multi-scan *SADABS2014*/5 (Bruker,2014/5) was used for absorption correction. wR2(int) was 0.1941 before and 0.0918 after correction. The Ratio of minimum to maximum transmission is 0.4400. The λ/2 correction factor is 0.00150.	Multi-scan *SADABS2014*/5 (Bruker,2014/5) was used for absorption correction. wR2(int) was 0.1999 before and 0.0963 after correction. The Ratio of minimum to maximum transmission is 0.3490. The λ/2 correction factor is 0.00150.
T_{min}, T_{max}	0.298, 0.746	0.274, 0.746	0.328, 0.746	0.260, 0.746
No. of measured, independent and observed [$I > 2\sigma(I)$] reflections	2088, 113, 107	2006, 112, 107	1871, 110, 106	1844, 108, 104
R_{int}	0.062	0.061	0.060	0.058
$(\sin \theta/\lambda)_{max}$ (Å^{-1})	0.753	0.750	0.747	0.748
Refinement				
$R[F^2 > 2\sigma(F^2)]$, $wR(F^2)$, S	0.024, 0.067, 1.16	0.025, 0.066, 1.23	0.032, 0.065, 1.26	0.028, 0.064, 1.29
No. of reflections	113	112	110	108
No. of parameters	7	7	7	7
$\Delta\rangle_{max}$, $\Delta\rangle_{min}$ (e Å^{-3})	0.09, -0.07	0.11, -0.10	0.13, -0.19	0.17, -0.14

Computer programs: *SAINT* v8.34A (Bruker, 2013), XL (Sheldrick, 2008), Olex2 (Dolomanov *et al.*, 2009).

Table S4. Experimental details

For all structures: Cubic, $Pa\bar{3}$, $Z = 4$. Experiments were carried out as detailed in Table S2. Refinement was on 9 parameters with 3 restraints.

	01_172k_2575	02_164k_2575	03_156k_2575	04_148k_2575
Crystal data				
Chemical formula	$0.18(N_2O).0.82(CO_2)$	$0.19(N_2O).0.81(CO_2)$	$0.18(N_2O).0.82(CO_2)$	$0.18(N_2O).0.82(CO_2)$
M_r	44.01	44.01	44.05	44.01
a (Å)	5.6952 (16)	5.6856 (15)	5.6784 (14)	5.6694 (14)
V (Å^3)	184.73 (15)	183.79 (14)	183.10 (13)	182.23 (13)
Radiation type	Synchrotron, λ = 0.6889 Å	Synchrotron, λ = 0.6889 Å	Synchrotron, λ = 0.6889 Å	Synchrotron, λ = 0.6889 Å
μ (mm^{-1})	0.15	0.15	0.15	0.16
Crystal size (mm)	0.5 × 0.28 × 0.28	0.5 × 0.28 × 0.28	0.5 × 0.28 × 0.28	0.5 × 0.28 × 0.28
Data collection				
Absorption correction	Multi-scan *SADABS2014*/5 (Bruker,2014/5) was used for absorption correction. wR2(int) was 0.1576 before and 0.0576 after correction. The Ratio of minimum to maximum transmission is 0.6088. The λ/2 correction factor is 0.00150.	Multi-scan *SADABS2014*/5 (Bruker,2014/5) was used for absorption correction. wR2(int) was 0.1474 before and 0.0732 after correction. The Ratio of minimum to maximum transmission is 0.5788. The λ/2 correction factor is 0.00150.	Multi-scan *SADABS2014*/5 (Bruker,2014/5) was used for absorption correction. wR2(int) was 0.1542 before and 0.0727 after correction. The Ratio of minimum to maximum transmission is 0.6055. The λ/2 correction factor is 0.00150.	Multi-scan *SADABS2014*/5 (Bruker,2014/5) was used for absorption correction. wR2(int) was 0.1516 before and 0.0703 after correction. The Ratio of minimum to maximum transmission is 0.7724. The λ/2 correction factor is 0.00150.
T_{min}, T_{max}	0.454, 0.746	0.432, 0.746	0.452, 0.746	0.576, 0.746
No. of measured, independent and observed [$I > 2\sigma(I)$] reflections	2877, 115, 90	2452, 111, 95	2416, 111, 97	2393, 111, 97
R_{int}	0.040	0.051	0.048	0.046
$(\sin \theta/\lambda)_{max}$ (Å^{-1})	0.755	0.751	0.752	0.754
Refinement				
$R[F^2 > 2\sigma(F^2)]$, $wR(F^2)$, S	0.039, 0.111, 1.21	0.024, 0.078, 1.27	0.022, 0.059, 1.16	0.024, 0.074, 1.16
No. of reflections	113	111	111	111
$\Delta\rangle_{max}$, $\Delta\rangle_{min}$ (e Å^{-3})	0.08, -0.09	0.04, -0.04	0.04, -0.05	0.04, -0.07

	05_140k_2575	06_132k_2575	07_124k_2575	08_116k_2575
Crystal data				
Chemical formula	0.19(N_2O).0.81(CO_2)	0.17(N_2O).0.83(CO_2)	0.12(N_2O).0.88(CO_2)	0.16(N_2O).0.84(CO_2)
M_r	44.01	44.01	44.01	44.05
a (Å)	5.6589 (13)	5.6499 (13)	5.6418 (12)	5.6345 (12)
V (Å^3)	181.22 (12)	180.35 (12)	179.58 (11)	178.88 (11)
Radiation type	Synchrotron, λ = 0.6889 Å	Synchrotron, λ = 0.6889 Å	Synchrotron, λ = 0.6889 Å	Synchrotron, λ = 0.6889 Å
μ (mm^{-1})	0.16	0.16	0.16	0.16
Crystal size (mm)	0.5 × 0.28 × 0.28	0.5 × 0.28 × 0.28	0.5 × 0.28 × 0.28	0.5 × 0.28 × 0.28
Data collection				
Absorption correction	Multi-scan *SADABS2014*/5 (Bruker,2014/5) was used for absorption correction. wR2(int) was 0.1484 before and 0.0786 after correction. The Ratio of minimum to maximum transmission is 0.6153. The λ/2 correction factor is 0.00150.	Multi-scan *SADABS2014*/5 (Bruker,2014/5) was used for absorption correction. wR2(int) was 0.1487 before and 0.0755 after correction. The Ratio of minimum to maximum transmission is 0.6001. The λ/2 correction factor is 0.00150.	Multi-scan *SADABS2014*/5 (Bruker,2014/5) was used for absorption correction. wR2(int) was 0.1481 before and 0.0730 after correction. The Ratio of minimum to maximum transmission is 0.6471. The λ/2 correction factor is 0.00150.	Multi-scan *SADABS2014*/5 (Bruker,2014/5) was used for absorption correction. wR2(int) was 0.1543 before and 0.0720 after correction. The Ratio of minimum to maximum transmission is 0.5582. The λ/2 correction factor is 0.00150.
T_{min}, T_{max}	0.459, 0.746	0.448, 0.746	0.483, 0.746	0.417, 0.746
No. of measured, independent and observed [$I > 2\sigma(I)$] reflections	2380, 110, 100	2376, 110, 99	2348, 110, 99	2332, 110, 100
R_{int}	0.052	0.050	0.050	0.051
$(\sin \theta/\lambda)_{max}$ (Å^{-1})	0.750	0.751	0.752	0.753
Refinement				
$R[F^2 > 2\sigma(F^2)]$, $wR(F^2)$, S	0.023, 0.072, 1.19	0.023, 0.063, 1.15	0.022, 0.050, 1.23	0.021, 0.055, 1.22
No. of reflections	110	110	110	110
$\Delta\rangle_{max}$, $\Delta\rangle_{min}$ (e Å^{-3})	0.04, -0.05	0.05, -0.06	0.07, -0.06	0.07, -0.06

	09_108k_2575	10_100k_2575
Crystal data		
Chemical formula	$0.18(N_2O).0.82(CO_2)$	$0.16(N_2O).0.84(CO_2)$
M_r	44.01	44.01
a (Å)	5.6264 (11)	5.6141 (11)
V (Å^3)	178.11 (10)	176.95 (10)
Radiation type	Synchrotron, λ = 0.6889 Å	Synchrotron, λ = 0.6889 Å
μ (mm^{-1})	0.16	0.16
Crystal size (mm)	0.5 × 0.28 × 0.28	0.5 × 0.28 × 0.28
Data collection		
Absorption correction	Multi-scan *SADABS2014*/5 (Bruker,2014/5) was used for absorption correction. wR2(int) was 0.1486 before and 0.0728 after correction. The Ratio of minimum to maximum transmission is 0.6199. The λ/2 correction factor is 0.00150.	Multi-scan *SADABS2014*/5 (Bruker,2014/5) was used for absorption correction. wR2(int) was 0.1497 before and 0.0795 after correction. The Ratio of minimum to maximum transmission is 0.5452. The λ/2 correction factor is 0.00150.
T_{min}, T_{max}	0.463, 0.746	0.407, 0.746
No. of measured, independent and observed [$I > 2\sigma(I)$] reflections	2328, 108, 98	2305, 108, 97
R_{int}	0.048	0.052
$(\sin \theta/\lambda)_{max}$ (Å^{-1})	0.744	0.745
Refinement		
$R[F^2 > 2\sigma(F^2)]$, $wR(F^2)$, S	0.021, 0.054, 1.22	0.021, 0.057, 1.18
No. of reflections	108	108
$\Delta\rangle_{max}$, $\Delta\rangle_{min}$ (e Å^{-3})	0.06, -0.04	0.06, -0.05

Computer programs: *SAINT* v8.34A (Bruker, 2013), XL (Sheldrick, 2008), Olex2 (Dolomanov *et al.*, 2009).

Table S5. Experimental details

For all structures: Cubic, $Pa\bar{3}$, $Z = 4$. Experiments were carried out as detailed in Table S2. Refinement was on 9 parameters with 3 restraints.

	01_172k_5050	02_164k_5050	03_156k_5050	04_148k_5050
Crystal data				
Chemical formula	$0.67(N_2O).0.33(CO_2)$	$0.65(N_2O).0.35(CO_2)$	$0.65(N_2O).0.35(CO_2)$	$0.65(N_2O).0.35(CO_2)$
M_r	44.02	44.02	43.17	43.98
Temperature (K)	172	164	156	148
a (Å)	5.7427 (13)	5.7327 (13)	5.7237 (12)	5.7144 (12)
V (Å^3)	189.39 (13)	188.40 (13)	187.51 (12)	186.60 (12)
μ (mm^{-1})	0.14	0.14	0.14	0.15
Crystal size (mm)	0.5 × 0.28 × 0.28	0.5 × 0.28 × 0.28	0.5 × 0.28 × 0.28	0.5 × 0.28 × 0.28
Data collection				
Absorption correction	Multi-scan *SADABS2014*/5 (Bruker,2014/5) was used for absorption correction. wR2(int) was 0.1125 before and 0.0484 after correction. The Ratio of minimum to maximum transmission is 0.7598. The λ/2 correction factor is 0.00150.	Multi-scan *SADABS2014*/5 (Bruker,2014/5) was used for absorption correction. wR2(int) was 0.1463 before and 0.0710 after correction. The Ratio of minimum to maximum transmission is 0.7671. The λ/2 correction factor is 0.00150.	Multi-scan *SADABS2014*/5 (Bruker,2014/5) was used for absorption correction. wR2(int) was 0.1487 before and 0.0676 after correction. The Ratio of minimum to maximum transmission is 0.7534. The λ/2 correction factor is 0.00150.	Multi-scan *SADABS2014*/5 (Bruker,2014/5) was used for absorption correction. wR2(int) was 0.1359 before and 0.0679 after correction. The Ratio of minimum to maximum transmission is 0.7711. The λ/2 correction factor is 0.00150.
T_{min}, T_{max}	0.567, 0.746	0.572, 0.746	0.562, 0.746	0.575, 0.746
No. of measured, independent and observed [$I > 2\sigma(I)$] reflections	2490, 114, 105	2460, 112, 102	2450, 112, 105	2428, 110, 104
R_{int}	0.039	0.051	0.048	0.049
$(\sin \theta/\lambda)_{max}$ (Å^{-1})	0.754	0.750	0.751	0.753
Refinement				
$R[F^2 > 2\sigma(F^2)]$, $wR(F^2)$, S	0.027, 0.073, 1.23	0.022, 0.057, 1.20	0.023, 0.059, 1.18	0.022, 0.055, 1.14
No. of reflections	114	112	112	110
$\Delta\rangle_{max}$, $\Delta\rangle_{min}$ (e Å^{-3})	0.04, -0.03	0.03, -0.04	0.05, -0.04	0.04, -0.03

	05_140k_5050	06_132k_5050	07_124k_5050	08_116k_5050
Crystal data				
Chemical formula	0.65(N_2O).0.35(CO_2)	0.65(N_2O).0.35(CO_2)	0.65(N_2O).0.35(CO_2)	0.65(N_2O).0.35(CO_2)
M_r	44.02	43.98	44.02	44.02
Temperature (K)	140	132	124	116
a (Å)	5.7057 (12)	5.6983 (12)	5.6917 (12)	5.6841 (11)
V (Å^3)	185.75 (12)	185.03 (12)	184.39 (12)	183.65 (11)
μ (mm^{-1})	0.15	0.15	0.15	0.15
Crystal size (mm)	0.5 × 0.28 × 0.28	0.5 × 0.28 × 0.28	0.5 × 0.28 × 0.28	0.5 × 0.28 × 0.28
Data collection				
Absorption correction	Multi-scan *SADABS2014*/5 (Bruker,2014/5) was used for absorption correction. wR2(int) was 0.1369 before and 0.0655 after correction. The Ratio of minimum to maximum transmission is 0.7593. The λ/2 correction factor is 0.00150.	Multi-scan *SADABS2014*/5 (Bruker,2014/5) was used for absorption correction. wR2(int) was 0.1425 before and 0.0635 after correction. The Ratio of minimum to maximum transmission is 0.7416. The λ/2 correction factor is 0.00150.	Multi-scan *SADABS2014*/5 (Bruker,2014/5) was used for absorption correction. wR2(int) was 0.1485 before and 0.0613 after correction. The Ratio of minimum to maximum transmission is 0.7395. The λ/2 correction factor is 0.00150.	Multi-scan *SADABS2014*/5 (Bruker,2014/5) was used for absorption correction. wR2(int) was 0.1365 before and 0.0613 after correction. The Ratio of minimum to maximum transmission is 0.7439. The λ/2 correction factor is 0.00150.
T_{min}, T_{max}	0.567, 0.746	0.553, 0.746	0.552, 0.746	0.555, 0.746
No. of measured, independent and observed [$I > 2\sigma(I)$] reflections	2423, 110, 105	2413, 109, 104	2405, 110, 105	2384, 109, 104
R_{int}	0.049	0.044	0.045	0.043
$(\sin \theta/\lambda)_{max}$ (Å^{-1})	0.754	0.755	0.756	0.752
Refinement				
$R[F^2 > 2\sigma(F^2)]$, $wR(F^2)$, S	0.027, 0.062, 1.16	0.023, 0.060, 1.12	0.022, 0.059, 1.20	0.024, 0.054, 1.15
No. of reflections	110	109	110	109
$\Delta\rangle_{max}$, $\Delta\rangle_{min}$ (e Å^{-3})	0.06, -0.06	0.06, -0.04	0.05, -0.04	0.07, -0.03

	09_108k_5050	10_100k_5050
Crystal data		
Chemical formula	$0.65(N_2O).0.35(CO_2)$	$0.65(N_2O).0.35(CO_2)$
M_r	44.02	44.05
Temperature (K)	108	100
a (Å)	5.6775 (11)	5.6715 (12)
V (Å^3)	183.01 (11)	182.43 (12)
μ (mm^{-1})	0.15	0.15
Crystal size (mm)	0.5 × 0.28 × 0.28	0.5 × 0.28 × 0.28
Data collection		
Absorption correction	Multi-scan *SADABS2014*/5 (Bruker,2014/5) was used for absorption correction. wR2(int) was 0.1444 before and 0.0642 after correction. The Ratio of minimum to maximum transmission is 0.7573. The λ/2 correction factor is 0.00150.	Multi-scan *SADABS2014*/5 (Bruker,2014/5) was used for absorption correction. wR2(int) was 0.1493 before and 0.0629 after correction. The Ratio of minimum to maximum transmission is 0.7113. The λ/2 correction factor is 0.00150.
T_{min}, T_{max}	0.565, 0.746	0.531, 0.746
No. of measured, independent and observed [$I > 2\sigma(I)$] reflections	2366, 110, 105	2390, 111, 107
R_{int}	0.044	0.044
$(\sin \theta/\lambda)_{max}$ (Å^{-1})	0.752	0.753
Refinement		
$R[F^2 > 2\sigma(F^2)]$, $wR(F^2)$, S	0.027, 0.060, 1.16	0.029, 0.060, 1.18
No. of reflections	110	111
$\Delta\rangle_{max}$, $\Delta\rangle_{min}$ (e Å^{-3})	0.08, -0.05	0.09, -0.05

Computer programs: *SAINT* v8.34A (Bruker, 2013), XT (Sheldrick, 2015), XL (Sheldrick, 2008), Olex2 (Dolomanov *et al.*, 2009).

Table S6. Experimental details

For all structures: Cubic, $Pa\bar{3}$, $Z = 4$. Experiments were carried out as detailed in Table S2. Refinement was on 9 parameters with 3 restraints.

	01_172k_7525	02_164k_7525	03_156k_7525	04_148k_7525
Crystal data				
Chemical formula	0.87(N_2O).0.13(CO_2)	0.89(N_2O).0.11(CO_2)	0.86(N_2O).0.14(CO_2)	0.85(N_2O).0.15(CO_2)
M_r	43.98	44.05	43.98	44.02
Temperature (K)	172	164	156	148
a (Å)	5.7564 (16)	5.7505 (16)	5.7414 (16)	5.7309 (16)
V (Å^3)	190.74 (16)	190.16 (16)	189.26 (16)	188.22 (16)
μ (mm^{-1})	0.14	0.14	0.14	0.14
Crystal size (mm)	0.5 × 0.28 × 0.28	0.5 × 0.28 × 0.28	0.5 × 0.28 × 0.28	0.5 × 0.28 × 0.28
Data collection				
Absorption correction	Multi-scan *SADABS2014*/5 (Bruker,2014/5) was used for absorption correction. wR2(int) was 0.1493 before and 0.0606 after correction. The Ratio of minimum to maximum transmission is 0.7797. The λ/2 correction factor is 0.00150.	Multi-scan *SADABS2014*/5 (Bruker,2014/5) was used for absorption correction. wR2(int) was 0.1493 before and 0.0563 after correction. The Ratio of minimum to maximum transmission is 0.7900. The λ/2 correction factor is 0.00150.	Multi-scan *SADABS2014*/5 (Bruker,2014/5) was used for absorption correction. wR2(int) was 0.1492 before and 0.0560 after correction. The Ratio of minimum to maximum transmission is 0.7787. The λ/2 correction factor is 0.00150.	Multi-scan *SADABS2014*/5 (Bruker,2014/5) was used for absorption correction. wR2(int) was 0.1486 before and 0.0568 after correction. The Ratio of minimum to maximum transmission is 0.7533. The λ/2 correction factor is 0.00150.
T_{min}, T_{max}	0.582, 0.746	0.590, 0.746	0.581, 0.746	0.562, 0.746
No. of measured, independent and observed [$I > 2\sigma(I)$] reflections	2392, 113, 110	2400, 112, 109	2379, 111, 109	2359, 110, 108
R_{int}	0.040	0.039	0.040	0.039
$(\sin \theta/\lambda)_{max}$ (Å^{-1})	0.752	0.753	0.749	0.751
Refinement				
$R[F^2 > 2\sigma(F^2)]$, $wR(F^2)$, S	0.030, 0.082, 1.21	0.029, 0.069, 1.18	0.027, 0.068, 1.35	0.024, 0.064, 1.25
No. of reflections	113	112	111	110
$\Delta\rangle_{max}$, $\Delta\rangle_{min}$ (e Å^{-3})	0.04, -0.03	0.04, -0.04	0.05, -0.03	0.04, -0.03

	05_140k_7525	06_132k_7525	07_124k_7525	08_116k_7525
Crystal data				
Chemical formula	0.85(N_2O).0.15(CO_2)	0.84(N_2O).0.16(CO_2)	0.85(N_2O).0.15(CO_2)	0.85(N_2O).0.15(CO_2)
M_r	44.02	44.02	44.02	44.02
Temperature (K)	140	132	124	116
a (Å)	5.7216 (16)	5.7128 (16)	5.7051 (16)	5.6974 (16)
V (Å^3)	187.31 (16)	186.44 (16)	185.69 (16)	184.94 (16)
μ (mm^{-1})	0.14	0.14	0.14	0.15
Crystal size (mm)	0.5 × 0.28 × 0.28	0.5 × 0.28 × 0.28	0.5 × 0.28 × 0.28	0.5 × 0.28 × 0.28
Data collection				
Absorption correction	Multi-scan *SADABS2014*/5 (Bruker,2014/5) was used for absorption correction. wR2(int) was 0.1442 before and 0.0537 after correction. The Ratio of minimum to maximum transmission is 0.7813. The λ/2 correction factor is 0.00150.	Multi-scan *SADABS2014*/5 (Bruker,2014/5) was used for absorption correction. wR2(int) was 0.1467 before and 0.0534 after correction. The Ratio of minimum to maximum transmission is 0.7800. The λ/2 correction factor is 0.00150.	Multi-scan *SADABS2014*/5 (Bruker,2014/5) was used for absorption correction. wR2(int) was 0.1473 before and 0.0529 after correction. The Ratio of minimum to maximum transmission is 0.7589. The λ/2 correction factor is 0.00150.	Multi-scan *SADABS2014*/5 (Bruker,2014/5) was used for absorption correction. wR2(int) was 0.1499 before and 0.0538 after correction. The Ratio of minimum to maximum transmission is 0.7238. The λ/2 correction factor is 0.00150.
T_{min}, T_{max}	0.583, 0.746	0.582, 0.746	0.566, 0.746	0.540, 0.746
No. of measured, independent and observed [$I > 2\sigma(I)$] reflections	2354, 110, 108	2334, 109, 107	2321, 109, 107	2316, 109, 107
R_{int}	0.038	0.039	0.037	0.039
$(\sin \theta/\lambda)_{max}$ (Å^{-1})	0.752	0.753	0.754	0.755
Refinement				
$R[F^2 > 2\sigma(F^2)]$, $wR(F^2)$, S	0.022, 0.058, 1.26	0.021, 0.054, 1.21	0.021, 0.053, 1.25	0.021, 0.051, 1.20
No. of reflections	110	109	109	109
$\Delta\rangle_{max}$, $\Delta\rangle_{min}$ (e Å^{-3})	0.04, -0.03	0.05, -0.04	0.05, -0.03	0.06, -0.04

	09_108k_7525	10_100k_7525
Crystal data		
Chemical formula	$0.85(N_2O).0.15(CO_2)$	$0.86(N_2O).0.14(CO_2)$
M_r	43.98	44.05
Temperature (K)	108	100
a (Å)	5.6906 (16)	5.6834 (15)
V (Å^3)	184.28 (16)	183.58 (15)
μ (mm^{-1})	0.15	0.15
Crystal size (mm)	0.5 × 0.28 × 0.28	0.5 × 0.28 × 0.28
Data collection		
Absorption correction	Multi-scan *SADABS2014*/5 (Bruker,2014/5) was used for absorption correction. wR2(int) was 0.1441 before and 0.0552 after correction. The Ratio of minimum to maximum transmission is 0.7324. The λ/2 correction factor is 0.00150.	Multi-scan *SADABS2014*/5 (Bruker,2014/5) was used for absorption correction. wR2(int) was 0.1468 before and 0.0564 after correction. The Ratio of minimum to maximum transmission is 0.7129. The λ/2 correction factor is 0.00150.
T_{min}, T_{max}	0.547, 0.746	0.532, 0.746
No. of measured, independent and observed [$I > 2\sigma(I)$] reflections	2286, 107, 106	2267, 107, 106
R_{int}	0.041	0.040
$(\sin \theta/\lambda)_{max}$ (Å^{-1})	0.751	0.752
Refinement		
$R[F^2 > 2\sigma(F^2)]$, $wR(F^2)$, S	0.020, 0.051, 1.18	0.023, 0.053, 1.19
No. of reflections	107	107
$\Delta\rangle_{max}$, $\Delta\rangle_{min}$ (e Å^{-3})	0.04, -0.04	0.05, -0.04

Computer programs: *SAINT* v8.34A (Bruker, 2013), XT (Sheldrick, 2015), XL (Sheldrick, 2008), Olex2 (Dolomanov *et al.*, 2009).

Raman Spectroscopy

The high pressure sample was analysed using a Jobin-Yvon Labram and T64000 Raman spectrometers equipped with He-Ne (633nm), and Ar-ion (488nm) laser, respectively.

Theoretical methods

DFT-D geometry optimisations. All geometry optimisation calculations were performed using the plane-wave DFT code CASTEP, version 8.0.[7] THE PBE exchange-correlation functional augmented with the TS dispersion correction was used,[8, 9] along with ultra-soft pseudopotentials generated 'on-the-fly'[10] and a plane-wave basis set expressed to an energy cut-off of 650 eV, which demonstrated convergence to within 0.2 meV/atom. K-points were sampled on a Monkhorst-Pack grid[11] of minimum spacing 0.08 Å^{-1}. The convergence tolerances for force, ionic displacement, stress and energy were 0.05 eVÅ^{-1}, 0.001 Å, 0.1 GPa and 0.01 meV/atom, respectively.

Cluster expansion Hamiltonian

Table S7 Actual and predicted total energies, from DFT and the cluster expansion Hamiltonian expression, respectively, along with nearest neighbour contact information for the training set of 12 randomly assigned crystallographic models based on a 2x2x2 primitive supercell lattice.

Model #	$\sum_{i=1}^{i=16}\sum_{j=1}^{j=12}\sigma_i\sigma_j$	Etotal/eV (from DFT)	Etotal/eV (from cluster expansion Hamiltonian)
1	-64	-16226.58194513	-16226.58205
2	-16	-16226.58967234	-16226.59025
3	-8	-16226.59241010	-16226.59162
4	-8	-16226.59159916	-16226.59162
5	-8	-16226.59246438	-16226.59162
6	-8	-16226.59099912	-16226.59162
7	-40	-16226.58670894	-16226.58615
8	-16	-16226.58971888	-16226.59025
9	-24	-16226.58970947	-16226.58888
10	0	-16226.59506292	-16226.59298

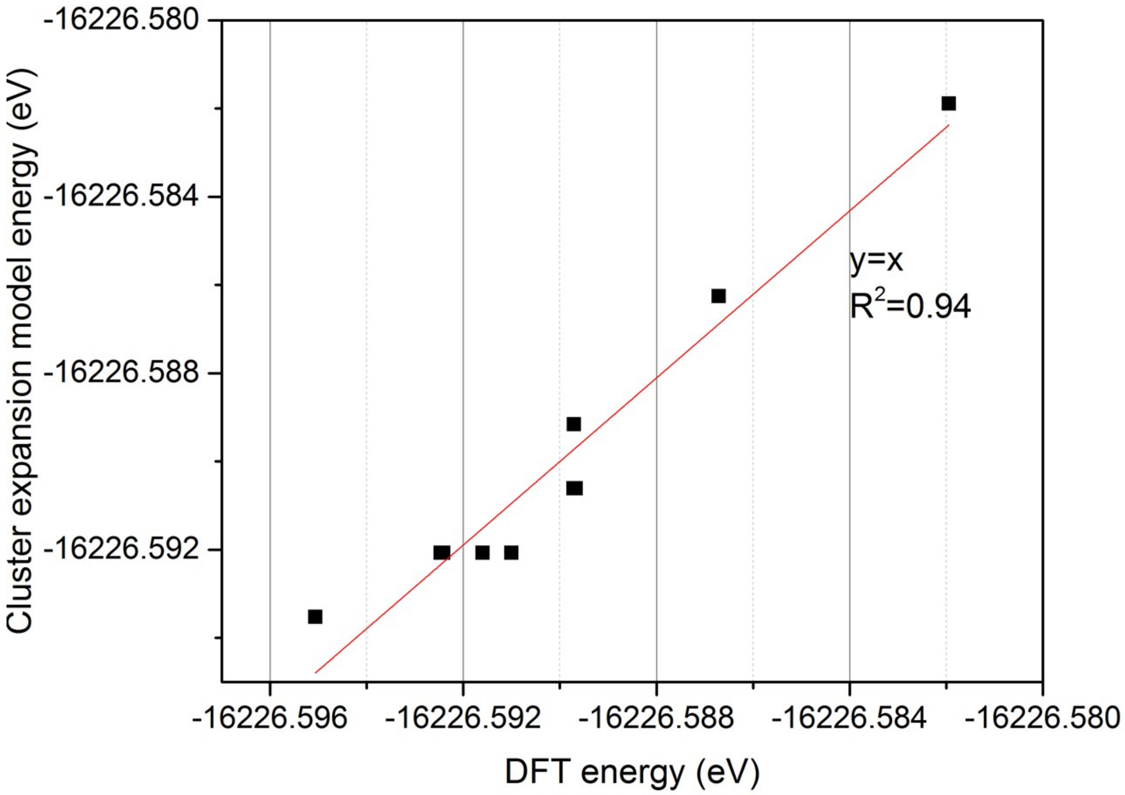

Figure S1 Correlation plot of actual total energy (derived from DFT calculations) versus predicted total energy (derived from cluster expansion Hamiltonian) for the training set of explicit 50:50 CO_2/N_2O crystal structures.

References

1. N. T. Johnson and M. J. Probert, *Journal*, 2016, DOI: https://github.com/nu-xtal-tools/cbf_to_sfrm.
2. G. Sheldrick, *Journal*, 2008, DOI: citeulike-article-id:8283092.
3. G. M. Sheldrick, *Acta Crystallographica a-Foundation and Advances*, 2015, **71**, 3-8.
4. O. V. Dolomanov, L. J. Bourhis, R. J. Gildea, J. A. K. Howard and H. Puschmann, *Journal of Applied Crystallography*, 2009, **42**, 339-341.
5. S. A. Moggach, D. R. Allan, S. Parsons and J. E. Warren, *Journal of Applied Crystallography*, 2008, **41**, 249-251.
6. G. J. Piermarini, S. Block, J. D. Barnett and R. A. Forman, *J. Appl. Phys.*, 1975, **46**, 2774-2780.
7. S. J. Clark, M. D. Segall, C. J. Pickard, P. J. Hasnip, M. J. Probert, K. Refson and M. C. Payne, *Zeitschrift Fur Kristallographie*, 2005, **220**, 567-570.
8. A. Tkatchenko and M. Scheffler, *Phys. Rev. Lett.*, 2009, **102**, 073005.
9. J. P. Perdew, J. A. Chevary, S. H. Vosko, K. A. Jackson, M. R. Pederson, D. J. Singh and C. Fiolhais, *Physical Review B*, 1992, **46**, 6671-6687.
10. D. Vanderbilt, *Physical Review B*, 1990, **41**, 7892-7895.
11. H. J. Monkhorst and J. D. Pack, *Physical Review B*, 1976, **13**, 5188-5192.
